# RNAi Knock-Down of LHCBM1, 2 and 3 Increases Photosynthetic H_2_ Production Efficiency of the Green Alga *Chlamydomonas reinhardtii*

**DOI:** 10.1371/journal.pone.0061375

**Published:** 2013-04-16

**Authors:** Melanie Oey, Ian L. Ross, Evan Stephens, Janina Steinbeck, Juliane Wolf, Khairul Adzfa Radzun, Johannes Kügler, Andrew K. Ringsmuth, Olaf Kruse, Ben Hankamer

**Affiliations:** 1 The University of Queensland, Institute for Molecular Bioscience, Brisbane, Queensland, Australia; 2 Institute of Plant Biology and Biotechnology, The University of Münster, Münster, Germany; 3 Center for Biotechnology (CeBiTec), Department of Algae Biotechnology & Bioenergy, Bielefeld University, Bielefeld, Germany; 4 Institute of Process Engineering in Life Sciences, Section II: Technical Biology, Karlsruhe Institute of Technology, Karlsruhe, Germany; 5 ARC Centre for Engineered Quantum Systems, The University of Queensland, Brisbane, Queensland, Australia; 6 Faculty of Applied Sciences, MARA University of Technology, Shah Alam, Selangor, Malaysia; Centro de Investigación y de Estudios Avanzados del IPN, Mexico

## Abstract

Single cell green algae (microalgae) are rapidly emerging as a platform for the production of sustainable fuels. Solar-driven H_2_ production from H_2_O theoretically provides the highest-efficiency route to fuel production in microalgae. This is because the H_2_-producing hydrogenase (HYDA) is directly coupled to the photosynthetic electron transport chain, thereby eliminating downstream energetic losses associated with the synthesis of carbohydrate and oils (feedstocks for methane, ethanol and oil-based fuels). Here we report the simultaneous knock-down of three light-harvesting complex proteins (LHCMB1, 2 and 3) in the high H_2_-producing *Chlamydomonas reinhardtii* mutant *Stm6Glc4* using an RNAi triple knock-down strategy. The resultant *Stm6Glc4L01* mutant exhibited a light green phenotype, reduced expression of *LHCBM1* (20.6% ±0.27%), *LHCBM2* (81.2% ±0.037%) and *LHCBM3* (41.4% ±0.05%) compared to 100% control levels, and improved light to H_2_ (180%) and biomass (165%) conversion efficiencies. The improved H_2_ production efficiency was achieved at increased solar flux densities (450 instead of ∼100 µE m^−2^ s^−1^) and high cell densities which are best suited for microalgae production as light is ideally the limiting factor. Our data suggests that the overall improved photon-to-H_2_ conversion efficiency is due to: 1) reduced loss of absorbed energy by non-photochemical quenching (fluorescence and heat losses) near the photobioreactor surface; 2) improved light distribution in the reactor; 3) reduced photoinhibition; 4) early onset of HYDA expression and 5) reduction of O_2_-induced inhibition of HYDA. The *Stm6Glc4L01* phenotype therefore provides important insights for the development of high-efficiency photobiological H_2_ production systems.

## Introduction

The development of clean fuels for the future is one of the most urgent challenges facing our society for three reasons; to reduce CO_2_ emissions, increase energy security and enable sustainable economic development. The importance of fuels is emphasized by the fact that, they directly supply ∼80% of global energy demand while electricity provides only ∼17% [1]. In terms of fuel security it is notable that in 2010 global energy use rose to ∼0.5 ZJ (1 ZJ = 10^21^ J) [2] and that the total global fuel reserves (oil, coal, gas and uranium) were reportedly at only ∼82 ZJ [3].

The capability to produce sufficient renewable fuel to supply global demand is dependent on the availability of a renewable energy source that is large enough to drive this process. Solar energy is by far the largest and most equitably distributed renewable energy source available, supplying ∼3020 ZJ yr^−1^ to the Earth’s surface [4]. Photosynthetically active radiation (PAR – conventionally 400≤ λ ≤700 nm [5]) makes up 43% of the irradiance delivered by the standard AM1.5 reference solar spectrum [6]. Therefore ∼1300 ZJ yr^−1^ is available globally to drive photosynthesis. Photosynthesis is a solar-to-chemical-energy conversion process that can be used to produce biomass and biofuels. Economic feasibility of photosynthetic fuel production systems is not limited by global solar irradiance, but rather by the areal productivities currently achievable cost-competitively. These depend strongly on the energy conversion efficiency of photosynthesis, which can be improved through engineering.

Microalgae systems are rapidly emerging as one of the most promising platforms for the production of renewable fuels and have a number of advantages compared to higher plants. In particular, they can theoretically address the key areas of concern related to the *food vs. fuel* conflicts of 1^st^ generation biofuel systems (e.g. corn ethanol) [7], [8] and assist with the development of a more sustainable *food and fuel* future. This is because well-designed microalgae systems can be located on non-arable land, cultivated at least in part using saline and waste water, enable increased nutrient recycling and achieve higher yields than crop plants, due to the ability to optimize light distribution, CO_2_ supply and production conditions [9]. Importantly, algae, besides being able to produce biomass and oil which can be converted into bio-fuels such as bio-diesel, methane and ethanol, also have the ability to produce hydrogen from water [10]. Solar powered H_2_ production is theoretically the most efficient method of biofuel production as it is closely coupled to the photosynthetic electron transport chain and additionally offers the potential to produce a CO_2_ - negative bio-fuel, reducing CO_2_ emission and assisting in carbon sequestration [11].

The first step of photosynthesis and all biofuel production is light capture. As a result, its optimization is essential for the development of all high-efficiency microalgal processes. In algal cells, light capture is performed by the chlorophyll-binding proteins of the Light Harvesting Complexes I (LHCI) and II (LHCII), which transfer the derived excitation energy to Photosystems I (PSI) and II (PSII), respectively. A secondary but very important role of the LHCII (LHCBM) proteins is that under high light conditions, they are involved in the photoprotective processes of non-photochemical quenching (NPQ) [12]–[15].

Given the need for microalgae to balance light capture and protection against photodamage, wild type algae (*wt*) have generally evolved large LHC antenna systems to capture incident light efficiently under low light conditions, and the ability to dissipate the excess (∼80%) under high light conditions to avoid photodamage [16]. *Wt* cells appear to have a competitive advantage, as they capture the bulk of the incident light at an illuminated surface and dissipate the excess, with the result that cells deeper in culture suffer from light limitation. Even though microalgae down-regulate LHCII levels naturally to a degree, under high light conditions, at 25% of full sunlight (full sunlight typically being 2000 µE m^−2^ s^−1^ midday at equatorial region) the photosynthetic capacity of most algal species is usually saturated [17] and energy dissipation is required. This can be broadly likened to a ‘*selfish organism strategy*’; i.e. each cell uses the light it needs and wastes the rest as heat, which cannot be used to drive photosynthesis in other cells.

The overall efficiency of the culture can theoretically be improved, by artificially reducing the light-harvesting antenna size. This strategy is designed to enable each cell to capture the light that it requires but allow the excess light that would otherwise be wasted, to penetrate further into the culture. This illuminates the cells that would otherwise be shaded. This strategy can be therefore broadly be likened to ‘*social engineering*’ in that the light is more evenly distributed between all cells in the culture. Thus, while the maximum light harvesting efficiency of individual cells should theoretically remain the same as long as the incident light exceeds saturating levels at the surface, the overall PBR efficiency can be increased. Due to the decreased energy wastage through NPQ, the concentration of algae cells can be increased, thereby improving the yield per unit volume of culture and reducing PBR costs. This offers a theoretical route to increase the efficiency and cost competitiveness of photo-biological fuel production.

In the green microalga *C. reinhardtii,* at least 25 LHC genes have been identified [18]. These exhibit a high degree of gene and protein sequence similarity across plant and algal families [14]. The LHCII proteins can be divided into the major and minor LHCII proteins. The major LHCII proteins are reportedly transcribed from 9 different genes *LHCBM 1–9* which are numbered according to the relative expression levels initially observed [19], [20]. These LHCBM proteins are reported to trimerize and their primary role is to capture solar energy and funnel it via the minor LHCII proteins, CP29 (*LHCB4*) and CP26 (*LHCB5*), to the PSII core (CP47, CP43, D1, D2, cytb559, PSBO and additional small and extrinsic subunits). In PSI solar energy is captured by nine LHCA proteins (LHCA1-9) and the derived excitation energy channeled to the PSI reaction center (psaA and psaB) [14], [21]–[23]. In higher plants the LHCI subunits reportedly form a crescent shaped belt on one side of the PSI monomer. This is termed the “LHCI-belt”, and reportedly consists of two dimeric subcomplexes. These subcomplexes are referred as LHCI-730 and LHCI-680 in higher plants and LHCI-705 and LHCI-680 in *Chlamydomonas*, where the numbers refer to their characteristic fluorescence peaks [24], [25].

Melis and coworkers and later others [16], [26]–[28] tested the hypothesis that artificial antenna reduction could improve light-to-biomass conversion efficiency and showed improvement under lab conditions. However, none of these strains were optimized for improved levels of H_2_ production in liquid culture.

Hydrogen production is typically induced by sulfur deprivation [10], [29]. Under these conditions the repair of the photo-inhibited D1 protein of PSII which contains methionine (and so S) is inhibited, reducing O_2_ production. This in turn lifts the inhibition of HYDA expression, inducing H_2_ production. Down regulation of PSII however also reduces the flow of H^+^ and e^-^ from H_2_O to H_2_.

Here we report the specific triple knock-down of the three most abundant LHCII proteins (LHCBM1, LHCBM2 and LHCBM3) in the high H_2_ producing background of *Stm6Glc4* [30], with the aim of further increasing the efficiency of photobiological H_2_ production. *Stm6Glc4* is based on the *Stm6* mutant, which under H_2_-producing conditions was shown to have upregulated Alternative Oxidase (AOX) activity [31] and to be locked into linear electron transport [32], resulting in increased O_2_ consumption and linear electron pathway from water to HYDA. The results show that reducing the LHC antenna system allows the rate of O_2_ production by photosynthesis to be brought into balance with the rate of O_2_ consumption by respiration. This in turn facilitates the induction of HYDA and provides a mechanism for continuous photosynthetic H_2_ production from water.

## Materials and Methods

### Strains and Culture Conditions

The high H_2_ producing *C. reinhardtii* strain *Stm6Glc4* described in [30] and *Stm6Glc4L01* were cultivated under mixotrophic (TAP (pH 7.2) [33] and photoautotrophic conditions (PCM (Photoautotrophic *Chlamydomonas* Medium) (pH 7, NH_4_Cl [30 mM], CaCl_2_*2H_2_0 [850 µM], MgSO_4_*3H_2_0 [1.5 mM], KH_2_PO_4_ [10 mM], FeSO_4_*7H_2_O [1 µM], CuSO_4_*5H_2_O [6.4 µM], MnCl_2_*4H_2_O [25.8 µM], ZnSO_4_*7H_2_O [77 µM], H_3_BO_3_ [184 µM], (NH_4_)_6_Mo_7_O_24_*4H_2_O [0.89 µM], CoCl_2_*6H_2_O [6.7 µM], Na_2_SeO_3_ [0.1 µM], VOSO_4_*H_2_O [0.009 µM], Na_2_SiO_3_*5H_2_O [273 µM], Na_2_EDTA [537.3 µM], Tris [100 mM], Vitamin B1 [52 µM], Vitamin B12 [0.1 µM]) under continuous white light (50 µE m^−2^ s^−1^), unless stated otherwise. Positive transformants were selected and cultivated under low light conditions (10 µE m^−2^ s^−1^) on TAP agar plates supplemented with 1.5 mM tryptophan, 60 µM 5-fluoroindole (5-FI) and hygromycin [30].

### Growth Rate µ_max_

To identify conditions yielding maximum microalgae growth rates in TAP and PCM, a number of factors were tested including light intensity (low light: 35 µE m^−2^ s^−1^ and high light: 450 µE m^−2^ s^−1^), culture volume (100, 112.5, 125, 137.5 and 150 µL trials corresponding to a culture depth of 2.7, 3, 3.5, 4 and 5 mm, respectively) and inoculation density (OD_750_ = 0.1 and 0.3). These experiments were conducted in a specially designed robotic Tecan Freedom Evo system which was fitted with three microwell shakers each capable of holding six 96 well microwell plates, a bank of fluorescent lights positioned 1.2 m from the microwell surface and a CO_2_ controller which maintained CO_2_ concentrations at 1±0.2% CO_2_ during photoautotrophic conditions. The lights consist of 12 cool white fluorescent lights (*Phillips PL-L55W/840 Cool White, Phillips International B.V. Netherland*) and 11 warm white fluorescent lights (*Phillips PL-L55W/830 Warm White, Phillips International B.V. Netherland*) which were installed in an alternating arrangement. The chamber temperature was 33±1°C. Algal cultures were adjusted to the desired OD_750_ (OD_750_ = 0.1 and 0.3, respectively) in TAP or PCM (supplemented with 1.5 mM tryptophan and 60 µM 5-FI for *Stm6Glc5L01*) and aliquoted into microwell plates. OD_750_ measurements were recorded at 3 h intervals using a dedicated spectrophotometer (Tecan Infinite M200 PRO microwell plate reader). Growth curves were generated using Microsoft Excel and Graph Pad Prism software was used for growth curve fitting. An R^2^ threshold of 0.85 was used to exclude false positives. The maximum value of the specific growth rate (µ_max_) was determined using a linear fit to ln OD_750_ vs. time, based on equation 1: µ = ln (ΔOD_750_)/Δt.

To exclude that the chosen 5-FI concentration of 60 µM impaired growth of the mutant strain, growth rate was monitored as described above in 150 µL TAP supplemented with tryptophan and different 5-FI concentrations (0, 30, 60, 90, 120, 240, 360, 480 µM) at 100 µE m^−2^ s^−1^.

### Biomass Determination

To determine direct biomass yields, *Stm6Glc4* and *Stm6Glc4L01* (grown in TAP medium) were adjusted to OD_750_ of 0.3 in 144 mL PCM (OD_750_ measurements were conducted using Tecan Freedom Evo system and a 96 well plate filled with 150 µL culture). Six 6 well microwell plates (total of 36 wells) for each strain were filled with 4 mL of adjusted cell suspension to a depth of 5 mm and incubated at a 1% CO_2_ atmosphere for 43 h with 450 µE m^−2^ s^−1^ illumination. OD_750_ was measured at the starting point and at 19 h and 43 h in the 6 well plates. After 43 h samples were harvested, pooled and washed in water to remove salt residues. Cells were dried at 60°C until no further mass change could be observed and obtained dry mass was calculated as g per Liter culture.

### Sequence Studies

All genome sequences were obtained from NCBI (National Center for Biotechnology Information, http://www.ncbi.nlm.nih.gov/, accessed March 2013) and analyzed using the BLAST tool provided by the *Chlamydomonas Center* (http://www.chlamy.org/, accessed March 2013). The cross BLAST analysis of all mRNA sequences against each other and the identification of non-homologous parts of the sequence led to a map of unique gene regions of the *LHCBM*. Linker and sense sequence of the inverted repeat construct were picked from these regions; the anti-sense region was determined using the “reverse complement” function of *Vector NTI* and confirmed by BLAST alignment.

### Plasmid Construction and Transformation

All nucleic acid work was conducted using standard procedures [34]. The *LHCBM1* target sequence was synthesized based on a 95 bp sequence of *LHCBM1* (bp 81–175), followed by an *Xba*I site. A subsequent 106 bp linker chosen from *LHCBM1* 3′UTR (bp 831–915) and the reverse orientated sequence of *LHCBM1* (bp 81–190) completed the RNAi target sequence. The *LHCBM2* target sequence was synthesized from two fragments. Fragment A consisted of a part of the *LHCBM2* 3′UTR (bp 1033–1132) followed by a *Sac*I site. Fragment B contained the reverse orientated sequence to fragment A (bp 1033–1160) with subsequent *EcoR*I and *Sac*I restriction sites. A linker region taken from *LHCBM2* (bp 11–103) with a preceding *Sac*I site and fragment B were amplified and joined via multiple template PCR. The linker and fragment B combination was cloned as a *Sac*I fragment into *Sac*I site downstream of fragment A to complete the inverted repeat sequence. The *LHCBM3* target sequence was synthesized from bp 628–728 of the *LHCBM3* 5′UTR followed by a *BamH*I site, a linker of *LHCBM3* 5′UTR (bp 255–404) and the reverse orientated 5′UTR sequence (bp 255–404) was synthesized. Additional cloning details are provided in the supplementary materials (Figure S1).The inverted repeat sequences were flanked by *EcoR*I restriction sites to facilitate subsequent cloning into the transformation vector *pBDH-R,* and yielded vectors *pMO52*, *pMO53* and *pMO75*, respectively. To create *pBDH-R* the Ribulose-1,5-bisphosphate-carboxylase/−oxygenase small subunit *(RBCS)* promoter, the intron fragment (with the start codon deleted) of *RBCS* was produced via fusion PCR and cloned into the *Xho*I and *Mlu*I site of *pALKK1* [27] to replace its *RBCS promoter/ATG/intron/BLE* region. This deletes an undesired *BLE* gene fragment present in *pALKK1*. To produce the triple knock-down strains, equal amounts of each vector *pMO52*, *pMO53* and *pMO75* were mixed and transformed into the strain *Stm6Glc4* [30] using biolistic bombardment [35].

### RNA Isolation, cDNA Synthesis and Quantitative Real-time PCR (qRT-PCR)

Isolation of total RNA was performed using a PureLink™ RNA Mini kit (Invitrogen) following the protocol optimized for animal and plant cells according to manufacturer’s directions. As *Stm6Glc4* is a cell wall deficient cell line the homogenization step was not carried out. DNase treatment was performed according to the PureLink™ RNA Mini kit manual using RQ1 RNase-free DNase (Promega). First strand cDNA synthesis was performed using iScript™ cDNA Synthesis kit (Bio-Rad Laboratories) and 1 µg total RNA according to manufacturer’s instructions. Quantitative real-time PCR analysis was carried out in triplicate using an Applied Biosystem 7500 Real Time PCR System with software SDS version 1.2.3 and the standard method ‘absolute quantification’ two step RT-PCR for thermal profile and dissociation stage (stage 1∶1 replication at 50°C, 1 min; stage 2∶1 replication at 95°C, 10 min; stage 3∶40 replications at 95°C, 15 sec; stage 4: dissociation; 1 replication at 95°C, 0.15 sec; 60°C, 1 min; 95°C, 0.15 sec). The experiment was carried out on a 96 well plate and each reaction contained 7.5 µl SYBR® Green Master Mix (Applied Bioscience), 5 µl of cDNA [1.6 ng µL^−1^], 2 µL of each primer [2 µM] and 3.5 µL of water. Relative expression levels were normalized using the *C. reinhardtii* reference gene *CBLP* [36]. Primer sequences for *LHCBM1*, *LHCBM2*, *LHCBM3* and *CBLP* were designed as reported previously [27], [36].

### FACS Analysis and Chlorophyll Measurements

Cells were grown to late log phase in TAP liquid culture (OD_750_ = ∼1.0), 1 mL of the culture was used for analysis in a FACSCanto II Flow Cytometer (Becton Dickson). Comparative chlorophyll fluorescence data was recorded using a 488 nm blue excitation laser and the 670 nm long pass detector to measure emission.

Total chlorophyll concentration was measured according to previously reported methods [37]. 1 mL of algae culture was sampled at late log phase (OD_750_ = ∼1.0). The cells were then pelleted (500×*g*, 5 min, 20°C) and the clear supernatant discarded before being resuspended in 1 mL of ice cold 80% acetone. After vortexing the sample, the precipitate was pelleted (14100×*g*, 4 min) before measuring the absorption (A) of the supernatant. The A_750_ was set to zero and A_664_ and A_647_ measured subsequently. Chlorophyll content was calculated as previously described [37].

### Demonstration of Algal Fluorescence at Different Depths

*Stm6Glc4L01* and *Stm6Glc4* were adjusted to same cell density (7.5*10^5^ cells mL^−1^) and transferred to a 100 mL glass cylinder positioned on a Safe Imager™ blue-light transilluminator (Invitrogen) fitted with light emitting diodes producing a narrow emission peak centered at ∼470 nm. Chlorophyll fluorescence was visualized by an ‘amber’ (530 nm long path) filter (Invitrogen).

### H_2_ Volume and Gas Composition Measurements

For H_2_ production *Stm6Glc4L01* and *Stm6Glc4* were grown to late log phase in TAP medium. Cells were harvested, washed twice with sulfur-free TAP medium and resuspended in sulfur-free TAP medium containing glucose [1 mM] [30] and adjusted to the same chlorophyll concentration (14.5 µg mL^−1^). H_2_ measurements were performed under continuous white light (450 µE m^−2^ s^−1^) using a custom-built PBR system with a dedicated gas collection tube mount [32]. The evolved gas was sampled using a gas-tight Hamilton syringe and injected at regular intervals into a gas chromatograph (Agilent Micro GC3000) fitted with a PlotU pre-column (3 m x 0.32 mm) and MolSieve 5APlot column (10 m x 0.32 mm). Argon (32.5 psi, pound per square inch) was used as the carrier gas and H_2_, O_2_ and N_2_ concentrations were monitored.

## Results

### Identification of *LHCBM* Target Sequences

**Figure 1:**
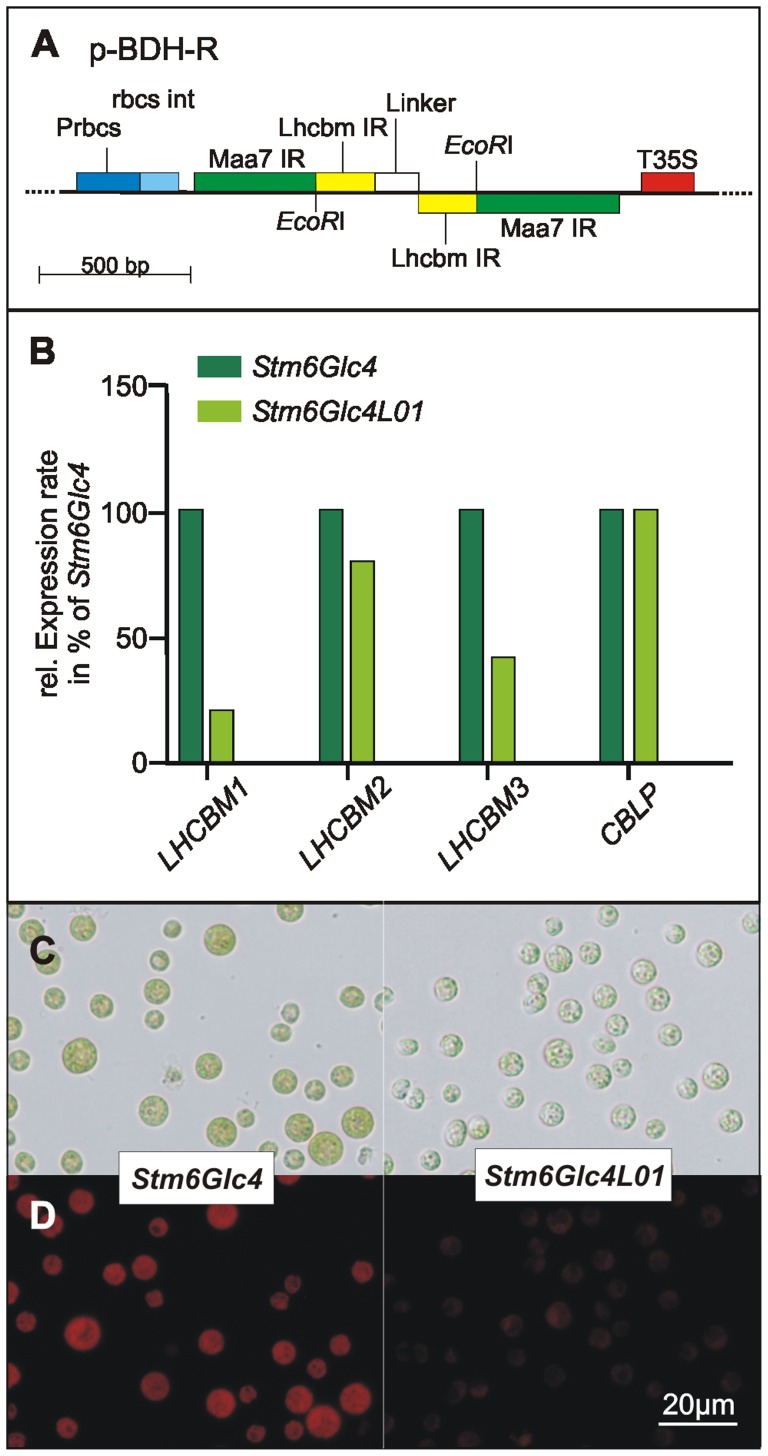
Schematic map of the transformation vector *pBDH-R*, relative abundance of LHC mRNAs and phenotypic cell distinction. (A) The RBCS promoter (Prbcs) with subsequent RBCS intron (rbcs int) and 35S terminator (T35S) flanking the RNAi expression cassette are marked. Sequences targeting the tryptophan synthase are indicated (Maa7 IR, [38]). Inverted repeat (IR) sequences used to target LHC genes (Lhcbm IR) and linker (Linker), which spaces the inverted repeats, are located in between two Maa7 inverted repeats (Maa7 IR). In this study ‘Lhcbm IR’ and ‘Linker’ were replaced with the sequences from the target *LHCBM* genes to minimize non-specific knock-down effects. *EcoR*I restriction sites used for cloning are marked. (B) mRNA levels of the three targeted LHCII genes (*LHCBM1* to *LHCBM3*) were determined in triplicate via quantitative real-time PCR and normalized to *CBLP* mRNA [36]. Expression levels (*LHCBM1*∶20.6±0.27; *LHCBM2*∶81.2±0.037 and *LHCBM3*∶41.4±0.05) were displayed as a percentage of the expression level of the parental strain *Stm6Glc4* (which was set to 100%). (C) Optical transmission microscopy of *Stm6Glc4* (left panel) and *Stm6Glc4L01* cells (right panel). (D) Chlorophyll autofluorescence image of *Stm6Glc4* (left panel) and *Stm6Glc4L01* cells (right panel) taken in an inverted fluorescence microscope (Nicon Ti-U) with identical settings.

To develop specific LHCBM knock-down strains, the identification of highly specific target sequences is essential. As all *LHCBM* genes display a high degree of sequence similarity [27], genomic BLAST analysis of the three target mRNAs, encoding the major light harvesting complex proteins of PSII (*LHCBM1, 2* and *3*), was performed. This analysis aimed to identify target regions that had minimal similarities to other genes and so prevent their co-suppression. Based on this analysis, discrete regions for each single *LHCBM* target (between 95–100 bp) were chosen for the development of RNAi constructs (sequences see Figure S1). To minimize non-specific RNAi effects further, the *LHCBM1-*, *2-* and *3*-specific RNAi constructs were engineered so that the linkers positioned between the sense and anti-sense regions of the construct were specific to the respective target genes. The plasmid *pALKK1* [27] was used to create the transformation vector *pBDH-R* (Figure 1A), additionally providing an RNAi target site for the endogenous tryptophan synthase. This allowed direct selection of positive transformants on media plates containing 5-fluoroindole (5-FI), as tryptophan synthase converts 5-FI into the toxic tryptophan analogue 5-fluorotryptophan [38].

### Development and Selection of Positive Transformants

Single *LHCBM* knock-out or knock-down transformants have not to date shown any marked phenotype in terms of chlorophyll concentration. This suggests that the knock-down of one LHCBM may be compensated for by the over expression of the remaining LHCBM proteins [39], [40]. Consequently a triple *LHCBM1*/*2*/*3* knock-down strategy was tested. LHCBM1, 2 and 3 were selected as they are reported to be the most abundant LHCII associated light harvesting proteins and so would be expected to achieve the greatest amount of antenna reduction [19], [20]. To develop triple knock-down mutants of *LHCBM1*, *LHCBM2* and *LHCBM3*, equal amounts of the target vectors were mixed and used to transform the high H_2_ producing *C. reinhardtii* strain *Stm6Glc4* [30] using biolistic bombardment [35]. Positive transformants were selected on agar plates containing 5-FI since, as based on the vector design, it was expected that transformants with knocked-down tryptophan synthase, and thus resistance to 5-FI, would co-suppress the desired LHC protein. This strategy enabled the testing of all possible combinations of *LHCBM1, LHCBM2* and *LHCBM3* knock-downs, with the low-antenna (light green) phenotype as the secondary selection criterion. Consistent with our hypothesis that the simultaneous knock-down of these major LHCs could result in a marked reduction in chlorophyll concentration, a number of light green *LCHBM1/2/3* multiple knock-down transformants were identified, of which the mutant with the palest phenotype (designated *Stm6Glc4L01* to signify *light harvesting mutant combination 01*) was chosen for further characterization.

### LHC mRNAs and Phenotypic Characterization

Total mRNA levels of *LHCBM1*, *LHCBM2* and *LHCBM3* in *Stm6Glc4L01* were determined using quantitative real-time PCR (qRT-PCR) to establish which of the target LHCBM genes were primarily affected. To confirm that equal amounts of cDNA were used in this experiment, data were compared with those of *18S* RNA and the mRNA for endogenous *C. reinhardtii* gene *CBLP* (*Chlamydomonas* ß-subunit-like polypeptide), which was previously shown to be stably expressed throughout growth and H_2_ production [36]. Data normalized to *CBLP* (Figure 1B) showed that *LHCBM1* has been down-regulated to 20.6% ±0.27% and *LHCBM3* to 41.4% ±0.05% of their original levels, respectively. The down-regulation of *LHCBM2* was less dramatic (81.2% ±0.037%) but importantly, *LHCBM2* levels did not increase to compensate for the knock-down of *LHCBM1* and *LHCBM3*. As gene dosage compensation has been suggested to be responsible for the unaffected phenotype of single antenna protein knock-outs [39]–[41], it is possible that the RNAi construct targeting *LHCBM2* functions primarily to limit its expression level, preventing such compensation. Data with absolute expression levels in addition to the fold-differences relative to *CBLP* (Figure 1B) are provided (Table S1). The similarity of expression levels for *18S* and *CBLP* genes between *Stm6Glc4* and *Stm6Glc4L01* suggests that the reduction in *LHCBM1* and -*3* levels is both meaningful and significant.

Light micrographs of *Stm6Glc4* and *Stm6Glc4L01* recorded under identical conditions (Figure 1C) showed that *Stm6Glc4L01* cells contained considerably lower chlorophyll levels than the *Stm6Glc4* parental control, consistent with the observed down-regulation of *LHCBM1*, *LHCBM2* and *LHCBM3* mRNA (Figure 1B) in a manner similar to that previously reported for other antenna mutants [16], [26], [27]. Auto-fluorescence images (Figure 1D), support the optical microscopy results and confirm that *Stm6Glc4L01* accumulates less chlorophyll per cell, as fluorescence levels are significantly lower than in *Stm6Glc4.* The increased chlorophyll a/b ratio (Mean: *Stm6Glc4*∶2.29±0.05 and *Stm6Glc4L01*∶2.62±0.09; *P*-value <0.0021 [37] is indicative of a specific reduction in the ratio of ChlB-rich LHC proteins compared with the ChlA-rich PSII core complexes [42] confirming LHC antenna size reduction.

### Fluorescence Properties and Chlorophyll Measurements

**Figure 2:**
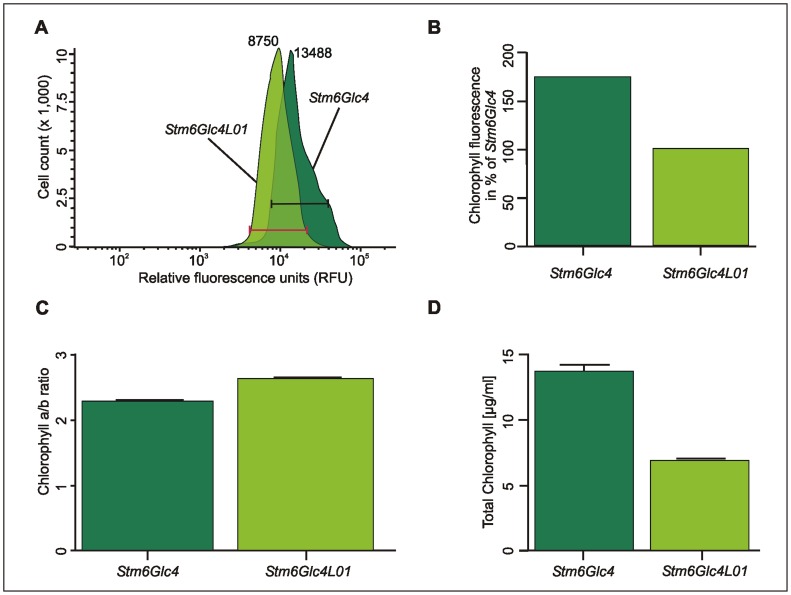
Chlorophyll fluorescence and chlorophyll yield in *Stm6Glc4* vs. *Stm6Glc4L01*. (A) Graph derived from flow cytometry analysis showing relative fluorescence units (RFU) per cell. Over 2.7*10^5^ cells were analyzed for each cell line. RFU range used to determine the mean is indicated by the red and black bars. (B) Mean chlorophyll fluorescence in percentage normalized to *Stm6Glc4L01* (100%). (C) Chlorophyll a/b ratio of *Stm6Glc4* and *Stm6Glc4L01.* (D) Total chlorophyll content in microgram per milliliter culture. Chlorophyll measurements (C, D) show results of two independent experiments with 7 replicates in total.

Flow cytometry (Figure 2A) was employed to quantify chlorophyll reduction in *Stm6Glc4L01* compared to the parental strain *Stm6Glc4*. For this purpose over 10^5^ cells (2.9×10^5^ cells for *Stm6Glc4*; 2.79×10^5^ cells for *Stm6Glc4L01*, grown under 50 µE m^−2^ s^−1^ illumination to late log phase) were analyzed and chlorophyll fluorescence compared (670 nm long pass filtered). The relative fluorescence of the *Stm6Glc4* culture peaked at 13488 relative fluorescence units (RFU) cell^−1^ compared to the 8750 RFU cell^−1^ of *Stm6Glc4L01*. Mean fluorescence was measured for *Stm6Glc4* at 15606 RFU cell^−1^ and for *Stm6Glc4L01* at 8951 RFU cell^−1^ (where the gate was set as indicated by the red and black lines in Figure 2A and displayed in Figure 2B as percentage of *Stm6Glc4L01* (100%). This suggests that the control cell line *Stm6Glc4* exhibited ∼175% higher levels of fluorescence losses than those observed in *Stm6Glc4L01* mutant (100%). Furthermore as the red fluorescence emission observed is due to the transition from the lower energy state of chlorophyll to the ground state, a concomitant heat loss is also be expected due to the transition from the higher to the lower energy state transition. Therefore *Stm6Glc4L01* is expected to display less heat losses than parental strain *Stm6Glc4*.

Though in practice, fluorescence measured by flow cytometry may not be strictly linear with respect to chlorophyll content [43], it indicates that *Stm6Glc4L01* exhibits a significant reduction compared to the parent strain. As the mean cell diameter of the two strains was similar (Figure 1C) a reduction in cell size cannot explain the reduced mean fluorescence in *Stm6Glc4L01*. The reduction in fluorescence in *Stm6Glc4LO1* is however consistent with the increased chlorophyll a/b ratio (*Stm6Glc4*∶2.29±0.05; *Stm6Glc4L01*∶2.62±0.09, Figure 2C) which is indicative of a reduction in the ratio of ChlB-rich LHC proteins compared with the ChlA-rich PSII core complexes [42] as well as the total reduction in chlorophyll concentration per cell (Figure 2D). The observation that the ChlA/B ratio has only increased from 2.29 to 2.62 is completely consistent with many independent observations including the following. The first is the fact that purified thylakoid membranes containing PSII-LHCII and PSI-LHCI typically have a ChlA/B ratio of about 1.9–2.3. The second is that purified PSII-core complexes from higher plants binding CP29, CP26 and CP24 (all of which bind ChlB), but which are almost completely devoid of LHCII and LHCI, have a ChlA/B ratio of ∼7 [44]. The fact that the ChlA/B ratio of *Stm6Glc4L01* cells is closer to 2.3 than 7 is therefore completely consistent with the fact that in addition to the ChlA/B binding proteins CP26 and CP29, the *Stm6Glc4L01* cells also contain the full complement of LHCA Chla/b proteins of which there are ∼9 different types, as well as LHCBM4-9 and residual levels of LHCBM1-3. Collectively this explains why the ChlA/B ratio of *Stm6Glc4L01* cells is significantly higher than that of the control (2.29+/−0.05 vs 2.62+/−0.09) but closer to 2.3 (*wt* thylokoids) than 7 (purified PSII core complexes).

Furthermore a determination of the total chlorophyll content [µg mL^−1^] showed that at the same cell number, total chlorophyll content was reduced by 50% in *Stm6Glc4L01* compared to *Stm6Glc4* (Figure 2D) (total chlorophyll content: *Stm6Glc4*∶13.65 µg mL^−1^, *Stm6Glc4L01*∶6.77 µg mL^−1^, *P*-value <0.0006.

### Light Penetration

**Figure 3:**
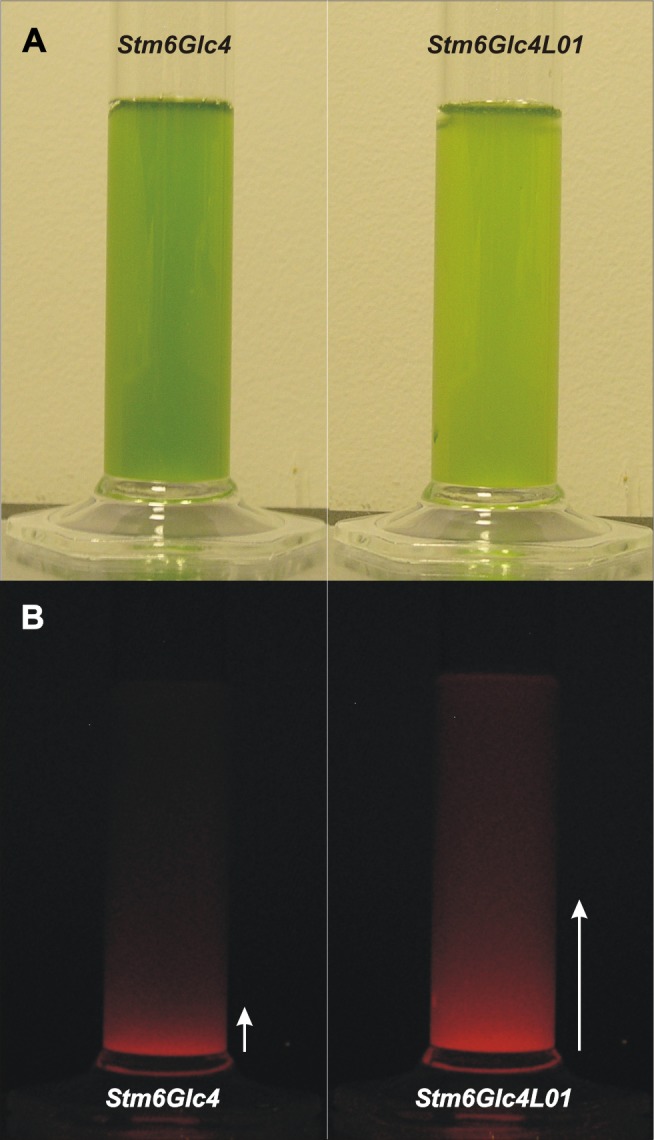
Light penetration into algal cultures with equal cell concentrations. Comparison of *Stm6Glc4* and *Stm6Glc4L01* algal cultures adjusted to the same cell number per mL in white light (A) and during illumination with blue light from below (B). Light penetration depth is indicated by white arrows. *Stm6Glc4L01* shows ∼4× more light penetration compared to parental strain *Stm6Glc4*.

To measure whether the decrease in LHCBM protein expression level increased light penetration through a liquid culture column, thereby making more light available for cells deeper in the culture, auto-fluorescence of *Stm6Glc4* and *Stm6Glc4L01* at the same cell density was measured (Figure 3A). As expected, *Stm6Glc4* cultures were a darker green color compared to those of *Stm6Glc4L01,* due to their larger LHC antenna. The cultures were then placed onto a blue light illuminator and chlorophyll auto-fluorescence imaged using an orange filter to block the blue background light. As illumination was provided from below, the height of the fluorescent column was indicative of the light penetration distance. The fluorescence throughout the *Stm6Glc4L01* culture column illustrates that light penetrated ∼4 times further than in *Stm6Glc4* at the same cell density (Figure 3B).

### Growth Rates Under Mixotrophic and Photoautotrophic Conditions

As light is both necessary and damaging for photosynthesis [45] as it is required to drive charge separation but in excess causes photoinhibition, the maximum rate of photochemistry is achieved at the point at which the photosystems are just saturated, assuming that light is the limiting factor. To minimize the area required for microalgae production and thereby also PBR costs, in theory systems should operate at the maximum flux density (high light) and the system poised at this optimal point by adjusting culture depth and cell density. Theoretically small antenna cell lines could allow more cells to be packed into a given unit volume before light becomes limiting and decrease both cell shading and photoinhibition. To test this hypothesis biomass production (as a proxy for chemical energy/biofuels) in form of maximum growth rates (µ_max_) of *Stm6Glc4* and *Stm6Glc4L01* cultures were measured under a range of culture depths and cell densities.

**Figure 4:**
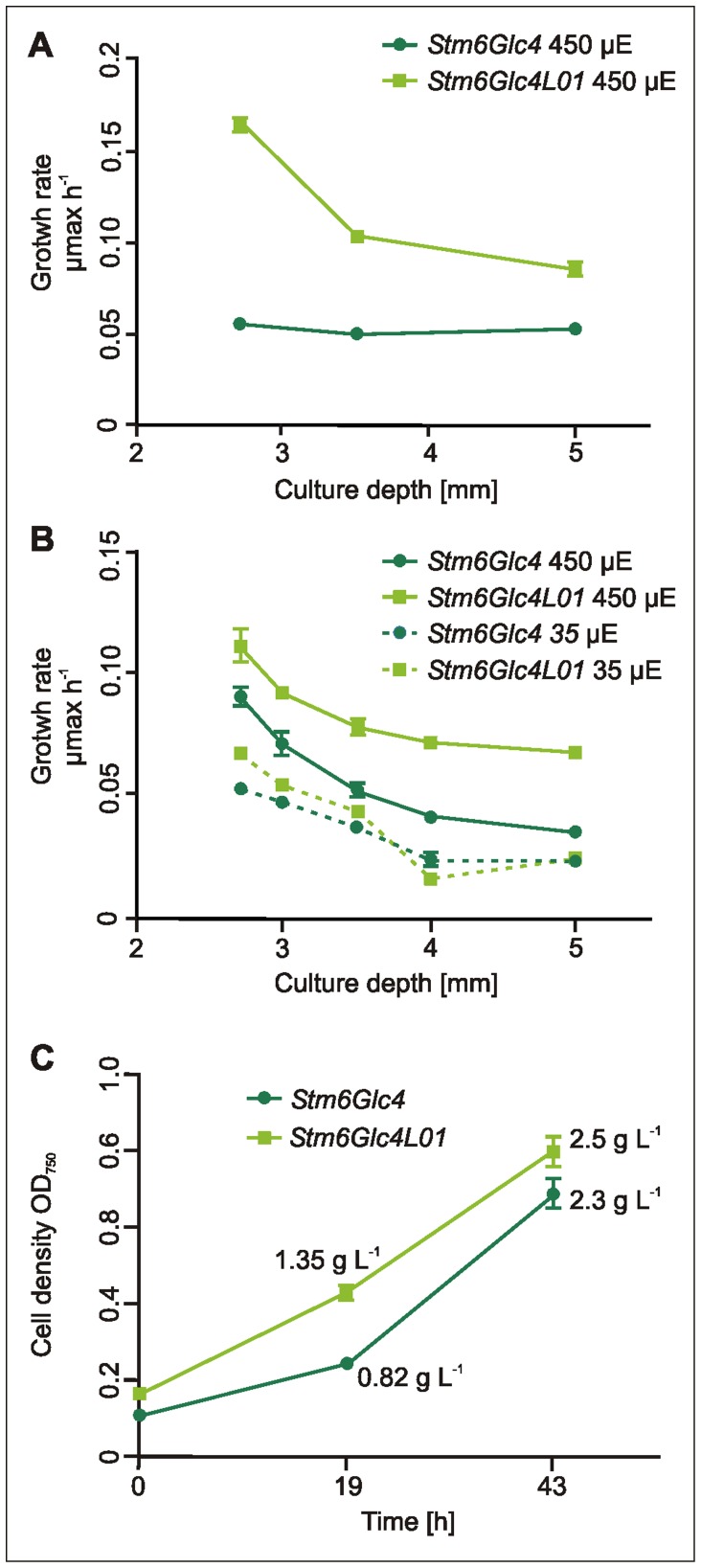
Growth rate µ_max_ h^−1^ of *Stm6Glc4* and *Stm6Glc4L01* at different cultivation conditions and biomass determination. (A) Growth rate at 450 µE m^−2^ s^−1^ under mixotrophic conditions at different culture depths using start OD_750_∶0.1. (B) Growth rate at 450 µE m^−2^ s^−1^ and 35 µE m^−2^ s^−1^under photoautotrophic conditions at different culture depths using start OD_750_∶0.3. Experimental data were compiled using triplicates. (C) OD_750_ and biomass determination in g L^−1^ under photoautotrophic conditions.

The growth rate of *Stm6Glc4* and *Stm6Glc4L01* was next determined under mixotrophic conditions (TAP medium) which are the best-established conditions for photobiological H_2_ production. Experiments were conducted in microwell plates using three different volumes (100 µL, 125 µL and 150 µL) to simulate different culture depths (average depths of 2.7, 3.5 and 5 mm, respectively). Since an advantage of *Stm6Glc4L01* was expected primarily in high light conditions, growth was measured under 450 µE m^−2^ s^−1^ illumination (optimal light intensity for the parental control and *wt* strains is 50–100 µE m^−2^ s^−1^) with an initial inoculation OD_750_ of 0.1. Under all conditions *Stm6Glc4L01* showed a higher growth rate than *Stm6Glc4* (Figure 4A). In the shortest path length cultures (100 µL = 2.7 mm) exposed to the highest light intensity (450 µE m^−2^ s^−1^) *Stm6Glc4L01* showed ∼3 times the growth rate of *Stm6Glc4* based on OD_750_ measurements. The increased growth rate could be due to reduced photoinhibition (due to the reduced LHC antenna size), improved light penetration and therefore increased total light-to-biomass conversion efficiency of the culture, or a combination of both factors. As the highest µ_max_ h^−1^ values for *Stm6Glc4L01* were observed in the shortest path length cultures (2.7 mm) and declined with increasing culture depth (Figure 4A) we concluded that path length and light distribution had a more dominant impact than photoinhibition. In contrast *Stm6Glc4* showed no such trend. It was previously reported [31] that *Stm6* (and hence *Stm6Glc4*) is light sensitive, demonstrated here by the poor growth displayed at very low cell densities, where no self-shading occurs at 450 µE m^−2^ s^−1^ (Figure 4A).

To investigate the growth behavior of *Stm6Glc4* and *Stm6Glc4L01* under photoautotrophic conditions, we performed a parallel experiment (Figure 4B) to determine the effects of culture depth (100 µL = 2.7 mm, 112.5 µL = 3 mm, 125 µL = 3.5 mm, 137.5 µL = 4 mm and 150 µL = 5 mm) at low (35 µE m^−2^ s^−1^) and high light levels (450 µE m^−2^ s^−1^). Previous experiments in TP medium (TAP medium minus acetate) indicated that N and P were limiting and led to an early culture growth plateau. Consequently for this experiment, an improved *in-house* medium was developed (Photoautotrophic *Chlamydomonas* Medium, PCM) which supplied more nitrogen and phosphate, to ensure that these did not limit growth. All cultures were inoculated at a starting OD_750_ of 0.3 to reduce light stress for *Stm6Glc4* at *450 *µE m^−2^ s^−1^.

At a culture depth of 2.7 mm and *450 *µE m^−2^ s^−1^ illumination, *Stm6Glc4L01* showed ∼120% of the maximum growth rate observed for the parental strain *Stm6Glc4*. Further increases in depth accentuated the advantage of the *Stm6Glc4L01* phenotype, as the growth rate at 5 mm depth was >185% compared to parental strain *Stm6Glc4*. This result shows that a reduction in antenna size improved growth across the conditions tested but particularly in deeper and denser cultures. In contrast, under low light levels (35**µE m^−2^ s^−1^) growth rates of *Stm6Glc4* and *Stm6Glc4L01* were comparable, indicating that the reduced antenna did not disadvantage the cells.

To confirm the OD_750_ measurements, direct biomass yields were also determined both for *Stm6Glc4* and *Stm6Glc4L01* grown in PCM under the best high light conditions tested (450 µE m^−2^ s^−1^, 5 mm culture depth, 1% CO_2_ atmosphere). OD_750_ was recorded at the starting point (0.113 for *Stm6Glc4*) and 0.169 for*Stm6Glc4L01*) and at time intervals (19 h and 43 h) (Figure 4C). To produce sufficient biomass to allow accurately biomass determination, samples were harvested at 43 h (OD_750_: *Stm6Glc4*∶0.692 g and *Stm6Glc4L01*∶0.803 g) and yielded 2.3 g L^−1^ (*Stm6Glc4*) and 2.5 g L^−1^ (*Stm6Glc4L01*) biomass dry weight, respectively. From this the biomass dry weight/OD_750 nm_ ratios of *Stm6Glc4* and *Stm6Glc4L01* were calculated to be 3.3 (2.3/0.692) and 3.1 (2.5/0.803) respectively. Based on these ratios, biomass yields at earlier time points were calculated Figure 4C. Although it appears initially that the differences in biomass yield at the 43 h time point are only approximately 10%, this was because the *Stm6Glc4L01* growth rate had started to plateau presumably due to exhausted nutrient resources. In contrast at the point of maximum growth (19 h) biomass concentrations were by this method calculated to be 0.818 g L^−1^ (*Stm6Glc4*) and 1.35 g L^−1^ (*Stm6Glc4L01*). This represents a 165% increase for *Stm6Glc4L01* over the control *Stm6Glc4* level (100%).

### Hydrogen Production

**Figure 5:**
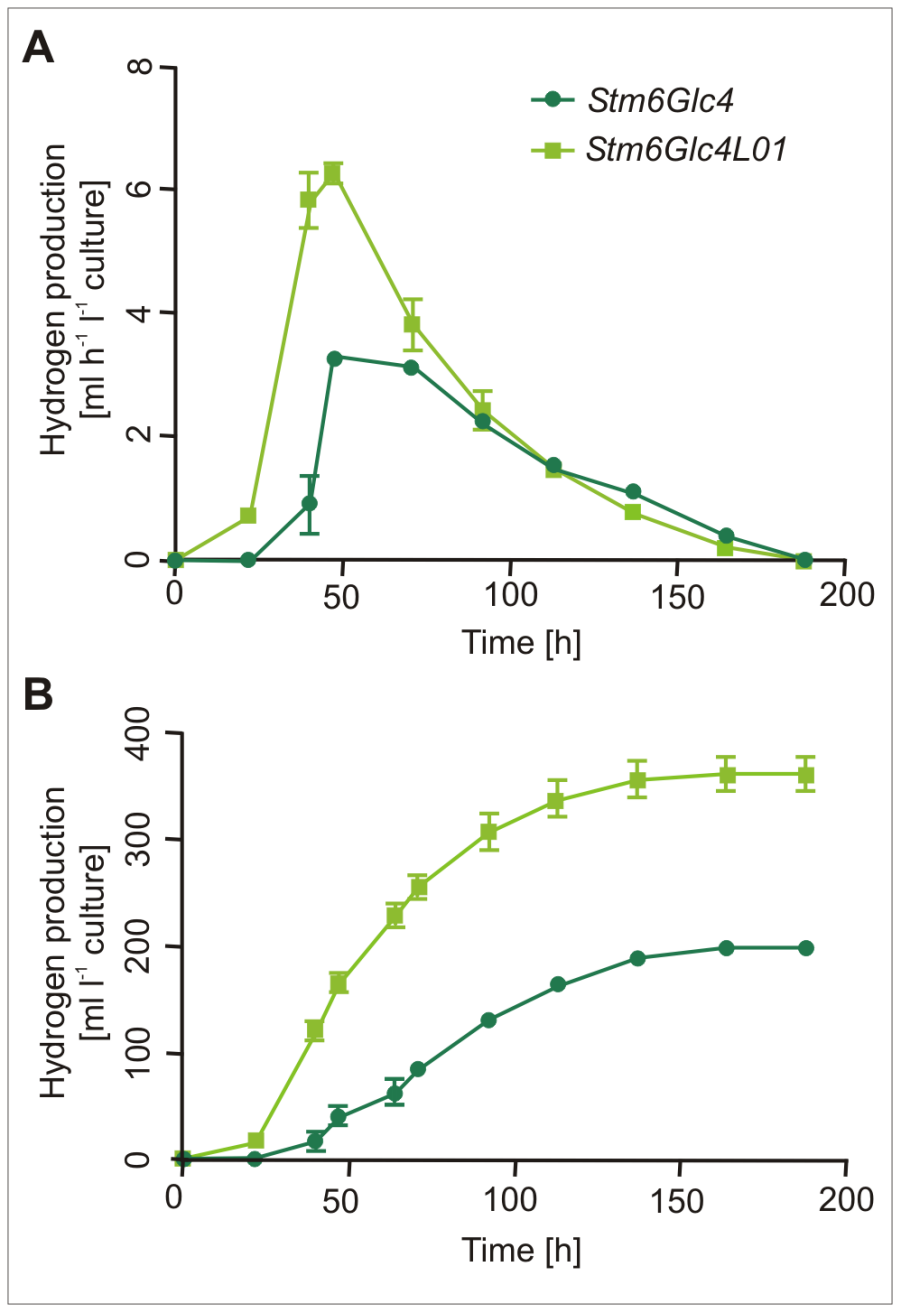
H_2_ production of *Stm6Glc4L01* and *Stm6Glc4*. H_2_ production rate in mL h^−1^ L^−1^ algae culture (A) and total H_2_ production in mL L^−1^ culture were determined (B). Experiments were performed under sulfur deprivation and with cultures adjusted to same chlorophyll content. Data were compiled using 3 replicates.

As photobiological H_2_ production is the most efficient light-to-biofuel conversion strategy [46] the effect of specific LHC gene down-regulation in *Stm6Glc4L01* on H_2_ generation was investigated. For this purpose a time course measuring H_2_ production rates (mL H_2_ L^−1^ h^−1^) in *Stm6Glc4* and *Stm6Glc4L01* cultures, adjusted to the same chlorophyll concentration (14.5 µg mL^−1^), was carried out in sulfur deprived media to establish anaerobiosis (Figure 5). At constant chlorophyll concentration and identical volumes, the cell number of *Stm6Glc4L01* was approximately double the cell number of *Stm6Glc4* (*Stm6Glc4*∶1×10^7^ cells mL^−1^; *Stm6Glc4L01*∶2×10^7^ cells mL^−1^). H_2_ production in the *Stm6Glc4L01* trials started almost immediately after transfer into sulfur deprived media in marked contrast to the ∼22 h lag time of the *Stm6Glc4* control (Figure 5A). This suggests that intracellular O_2_ concentrations were lower in *Stm6Glc4L01* than in *Stm6Glc4* and close to or below the induction threshold for HYDA expression. The reduced lag phase has marked operational benefits, and significantly increases the yield. The H_2_ production rates of both strains peaked at 47 h after sulfur deprivation, with *Stm6Glc4L01* producing 6.25 mL L^−1^ h^−1^ compared to 3.31 mL L^−1^ h^−1^ for *Stm6Glc4* under the conditions tested. Both strains produced H_2_ until 188 h after sulfur deprivation with a similar decline in both strains after peak production. Under the conditions tested *Stm6Glc4L01* produced a total volume of 361 mL ±27.6 mL H_2_ per liter culture compared to 198 mL ±21.2 mL H_2_ per liter culture produced by *Stm6Glc4. Stm6Glc4L01* therefore produced >180% more H_2_ per unit chlorophyll than the *Stm6Glc4* control (Figure 5B), which via gas chromatography was determined to be >95% pure. These results clearly show that *Stm6Glc4L01* exhibited improved characteristics in terms of early onset and higher rates of H_2_ production.

## Discussion

### Photosynthetic Efficiency

Light capture is the first step of all microalgae-based fuel production and its optimization is therefore of fundamental importance for the development of high-efficiency processes. Wild type microalgae antennae have evolved intricate mechanisms to adapt to natural fluctuations in light levels and quality to maximize light capture under low light conditions and dissipate excess light under high light conditions. While algae possess the ability to down-regulate their antenna, even 25% of full sunlight is sufficient to saturate the photosynthetic capacity of most algal species [17]. Under such over-saturated light conditions for a well-mixed culture in an open raceway or closed PBR, energy losses of up to 80% can occur [47]. This is because cells near the illuminated surface dissipate excess solar energy via NPQ which is then no longer available to cells deeper in the culture and which are therefore light-limited. Consequently, while these mechanisms are finely tuned for adaptation to their natural environment, they are far from optimized for industrial microalgae production, which to minimize area requirement and photobioreactor (PBR) construction costs should ideally be established for high flux density conditions. Photobioreactors can be designed to achieve light dilution (e.g. to 10% of 2000 µE m^−2^ s^−1^), but due to increased complexity and material costs this increases photobioreactor expense. Antenna engineering has the advantage that it can theoretically improve production efficiency and reduce capital costs both of which are major economic drivers for this technology [48].

Given the large number of LHC proteins in eukaryotic photosynthetic organisms, a diversity of function among family members is implied. The generation of *Stm3LR3* [27] involved knocking-down all LHC classes and resulted in a low chlorophyll phenotype. It was however recognized during this initial antenna engineering step that some vital LHC functions might also be affected. This assumption was borne out by subsequent work [49] and the observation that *Stm3LR3* is moderately light-sensitive at low cell densities. For example, CP29 (*LHCB4*) is essential for state transitions and for docking the LHCBM1, −2 and −3 trimers to PSI [49], while a role for CP26 (*LHCB5*) in preventing photoinhibition has also been described [13], [15], [49]–[51]. It is therefore likely that the pattern of LHC down-regulation displayed in *Stm3LR3* does not preserve all of the functions required to support growth at high light levels [40].

While the high degree of homology in LHCs reflects a common ancestry, it also suggests that specific functions likely reside in the small non-conserved regions. Consequently, precise engineering of specific LHC proteins is important to avoid undesired effects due to co-suppression of other genes. As PSII is the main site of light-induced damage, there is a strong correlation between PSII antenna size and photosynthetic productivity [47], [52]–[54]. This supports the idea that the most abundant LHCII antenna proteins, LHCBM1, −2 and −3, are the most important targets for down-regulation. In this context it is of note that while the exact molecular mechanisms for photodamage of PSII are still under investigation (for review see [54]), it has been shown that reduced chlorophyll content plays a crucial role in preventing photodamage, and thus allows the cells to grow under higher light conditions [55], supporting our findings. The above experiments show that LHCBM1 (20.6% ±0.27%), LHCBM2 (81.2% ±0.037%) and LHCBM3 (41.4% ±0.05%) expression levels were effectively knocked-down (Figure 1B), resulting in the light green mutant *Stm6Glc4L01* (Figure 1C), which had a reduced total chlorophyll content (50% of parental strain (Figure 2D)) and an increased ChlA/B ratio (*Stm6Glc4*∶2.29±0.05 vs. *Stm6Glc4L01*∶2.62±0.09) (Figure 2C), confirming LHCII depletion. Down-regulation of the LHCII antenna system was further supported by chlorophyll auto-fluorescence measurements obtained by flow cytometry (Figure 2A and B). Due to its reduced antenna size, cultures of *Stm6Glc4L01* exhibited considerably improved light penetration (Figure 3B) over those of the control (*Stm6Glc4*) at the same cell density. Our data also indicates that the improved light penetration is in part due to reduced fluorescence and heat losses at the illuminated culture surface (Figure 2B) and in part due to the reduced chlorophyll content of each cell (Figure 2D) which improves light transmission through the culture (Figure 3B). Together these properties resulted in an increased fraction of cells in the culture being poised close to the optimum point of light saturation (achieving maximal rates of photochemistry, without causing photo-inhibition), rather than within the broad range of illumination levels typically experienced by cells in the *Stm6Glc4* control culture. This improved poise was achieved at light levels of 450 µE m^−2^ s^−1^ instead of the more typical level of 100 µE m^−2^ s^−1^ at which *Stm6Glc4* antenna cell line is saturated and led to growth rates of >185% of *Stm6Glc4* under photoautotrophic conditions (Figure 4B). The importance of this is that under photoautotrophic conditions required for both biomass and ideally commercial scale H_2_ production, at outside light levels (e.g. 2000 µE m^−2^ s^−1^), the light dilution factor could be reduced from 2000/100 = 20 to 2000/450 = ∼4.4 [52], offering the potential for significant savings in photobioreactor costs.

### Molecular Mechanism of Improved H_2_ Production

**Figure 6:**
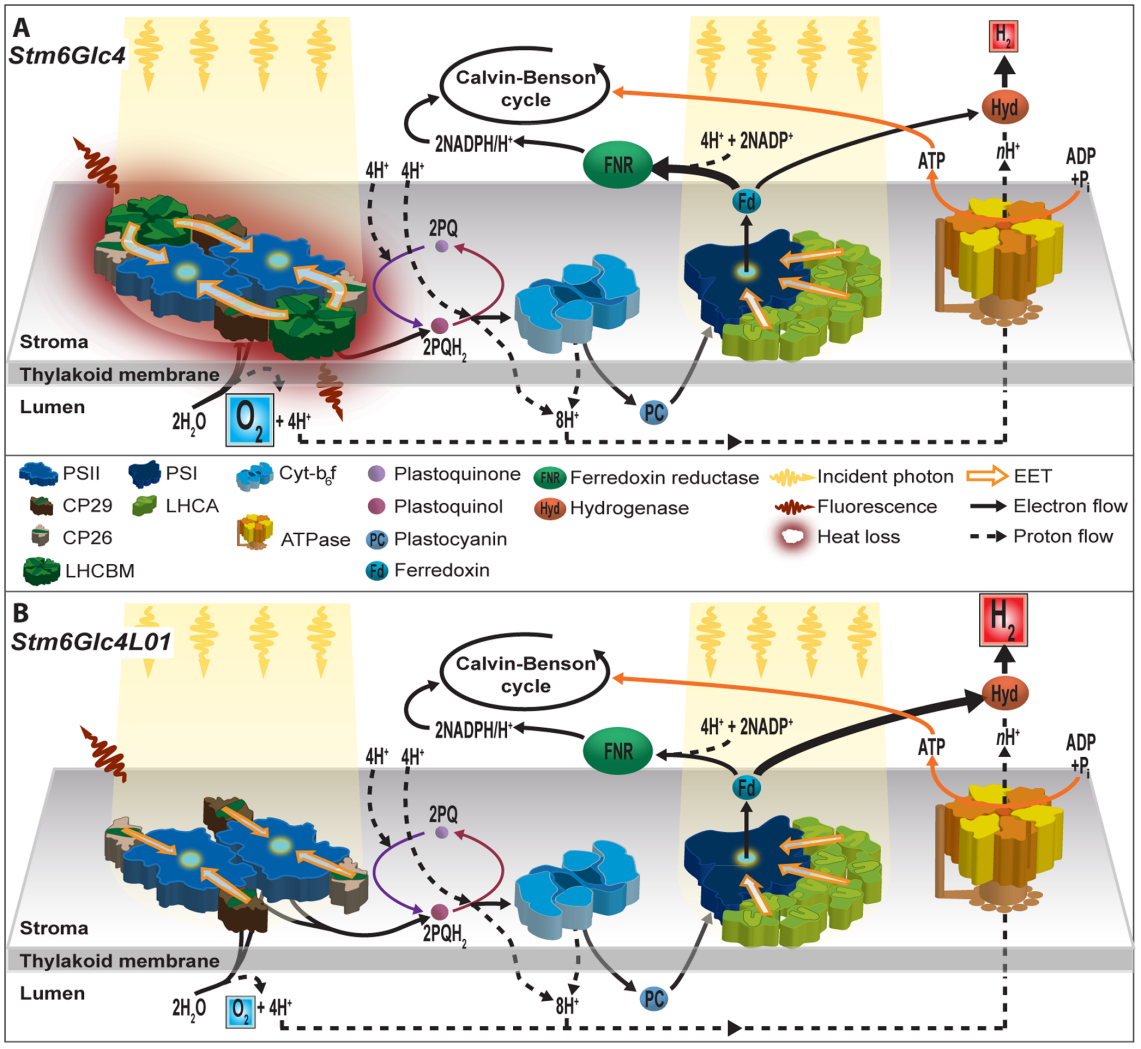
Mechanistic model of improved H_2_ production in *Stm6Glc4L01*. (A) *Stm6Glc4* has a large PSII antenna system consisting of LHCBM1-9. LHCBM1-3 are reported to be most abundant. Large antenna size results in increased PSII mediated O_2_ production and NPQ losses. NPQ losses reduce system efficiency; intracellular O_2_ levels inhibit expression of HYDA until the system is sulfur deprived (sulfur required for the repair of the PSII-D1 subunit. **B:** *Stm6Glc4L01* has a reduced antenna size which is figuratively shown, and leads to reduced O_2_ production and early onset of H_2_ production. The light green phenotype allows higher cell densities to be used leading to increased rates of H_2_ production.

The results presented are summarized with the following mechanistic model (Figure 6). In *wt* cells, *Stm6* and *Stm6Glc4*, light used by PSII is predominantly captured by LHCBM trimers bound to the PSII core complex. The major LHCII proteins are reportedly transcribed from 9 different genes LHCBM 1–9 which are numbered according to the relative expression levels initially observed (16, 17). These LHCBM proteins are reported to trimerize and their primary role is to capture solar energy and funnel it via the minor LHCII proteins, CP29 (LHCB4) and CP26 (LHCB5), to the PSII core (CP47, CP43, D1, D2, cytb559, PSBO and additional small and extrinsic subunits) (Figure 6). Down regulating LHCBM1-3 which are the most abundant of these, as expected, resulted in a marked reduction in antenna size. It should be noted that LHCBM1 has also been reported be to be involved in NPQ as well as the scavenging of radical oxygen species [40]. While the down regulation of these functions might be expected to have a detrimental effect at high light levels, our results indicate improved biomass and H_2_ yields at 450 μΕ m^−2^ s^−1^ (>100 µE m^−2^ s^−1^ can saturate *Stm6* and *Stm6Glc4*). This can be explained by the small antenna size of *Stm6Glc4L01* resulting in lower levels of over excitation of PSII. This in turn is expected to result in the reduction of the formation of radical oxygen species and the requirement for the dissipation of excess energy via NPQ. Consequently the down regulation of the NPQ and ROS scavenging functions through the knock-down of LHCBM1 appears to be compensated for by the reduction of antenna size.

The light captured by the PSII antenna system drives the PSII-mediated oxidation of water to H^+^, e^-^ and O_2_ (Figure 6A) [32]. Electron transport subsequently progresses via plastoquinone (PQ), cytochrome b_6_f, plastocyanin to PSI where electrons are again energized by light, to reduce ferredoxin (Fd). Ferredoxin nucleotide reductase (FNR) couples the reoxidation of Fd to the reduction of NADP^+^ which under normal aerobic conditions is then used during carbon fixation to reduce CO_2_ to sugars. During this process, protons are translocated to the lumen establishing the proton gradient which drives ATP production. Apart from water, products derived from starch metabolism can be used to provide electrons to PQ via the NADPH dehydrogenase (NDA2) [56]. Under illuminated anaerobic/micro-oxic conditions the derived H^+^ and e^-^ can also act as substrates for HYDA-mediated H_2_ production. It is of note however that up to 80% of the H^+^ and e^-^ used by HYDA are reportedly produced by PSII [57], providing a direct pathway for the solar driven H_2_ production from water.

H_2_ production requires both illumination (to drive photosynthesis) and anaerobic conditions (to induce the expression of HYDA and prevent HYDA inhibition) [10]. Under anaerobic conditions, mitochondrial respiration largely ceases as the cell is deprived of O_2_. The expression of HYDA however provides an alternative H^+^/e^-^ release mechanism which recombines H^+^ and e^-^ to produce H_2_ gas that is excreted from the cell (i.e. instead of H_2_O from mitochondria). This allows photosynthetic electron transport to continue to operate, providing an alternative pathway for the production of ATP and NADPH to enable cell survival (11).

In the *wt*, only small amounts of H_2_ are typically produced as the large antenna phenotype increases oxygen production in illuminated cells, which is not removed quickly enough by *wt* respiration levels and so leads to a reduction in HYDA transcription and function. Typically, anaerobiosis can be established in *wt* algae cultures through sulfur depletion. This is because the Methionine containing D1 protein of PSII, which is rapidly photo-damaged under high light conditions, can only be repaired in the presence of sulfur. Consequently, by limiting sulfur, PSII-mediated O_2_ evolution is gradually reduced over time. When it drops below the rate of O_2_ uptake by mitochondrial respiration, the system becomes micro-oxic or anaerobic and induces HYDA expression [58].

The parental strains of *Stm6Glc4L01* are *moc1* mutants (i.e. *Stm6* and *Stm6Glc4* [30]–[32]). Both have an increased respiration rate due to upregulated AOX activity (high O_2_ consumption), but also have a large *wt* like PSII antenna system (Figure 6A). As a result of their increased rate of respiration *Stm6* and *Stm6Glc4* exhibit a higher rate of oxygen consumption than the *wt* and so enter anaerobiosis faster under sulfur deprivation. This in turn results in earlier activation of HYDA expression [59] compared to the *wt* leading to the rapid onset of H_2_ production. However the relatively large antenna systems of *Stm6* and *Stm6Glc4* still supports rates of O_2_ production which exceed the respiration rate and so sulfur depletion is required to induce H_2_ production.

In contrast in S*tm6Glc4L01* the LHCBM1, LHCBM2 and LHCBM3 have been down-regulated within an upregulated AOX background (Figure 6B). The importance of this is that the simultaneous reduction of O_2_ production and increased O_2_ consumption is thought to reduce intracellular O_2_ levels below the induction threshold for HYDA. This hypothesis is supported by three observations. First, *Stm6Glc4L01* cultures showed an almost immediate onset of H_2_ production upon sulfur deprivation (Figure5A) indicating that the intracellular O_2_ levels in sulfur replete medium are already poised closed to HYDA threshold level. Second, during the early stages of H_2_ production the contaminating O_2_ concentrations in the gas produced by *Stm6Glc4LO1* cultures were considerably lower than those produced by *Stm6Glc4*. Third, the rate of H_2_ production by *Stm6Glc4L01* was almost twice as high as that observed for *Stm6Glc4* (Figure 5B) for a given culture volume adjusted to the same chlorophyll concentration, for reasons described above.

The finding that the *Stm6Glc4L01* is so closely poised to HYDA expression opens up the exciting possibility of using this mutant for continuous H_2_ production in sulfur replete medium. To date, to our knowledge continuous H_2_ production in sulfur replete liquid medium has not been reported, presumably because under these conditions O_2_ production usually exceeds O_2_ consumption inhibition HYDA expression. For example the strains *Stm3LR3* [27], *Stm6Glc4T7* [26] and *TLA1* [16], [60] have not been reported to increase H_2_ yield in liquid culture which would be desirable for the development of continuous H_2_ production in scale PBR systems. However, important steps along this development path include the use of sulfur microdosing approaches [61] and the use of alginate solid phase systems which reduced O_2_ inhibition [60].

In summary, *Stm6Glc4L01* exhibited an improved H_2_ and biomass production efficiency (165–180% improvement over parental strain). Importantly, this was achieved at increased solar flux densities (450 µE m^−2^ s^−1^) and high cell densities which are best suited for microalgae production as light should ideally be the limiting factor. Our data suggests that the overall improved photon to H_2_ conversion efficiency is due to: 1) reduced loss of absorbed energy by non-photochemical quenching (fluorescence and heat losses) near the photobioreactor surface; 2) improved light distribution in the reactor; 3) reduced photoinhibition; 4) early onset of HYDA expression, 5) reduction of O_2_ induced inhibition of HYDA. The *Stm6Glc4L01* phenotype therefore provides important insights for the development of high efficiency photobiological H_2_ production systems.

## Supporting Information

Figure S1 **Initial RNAi oligo-nucleotides used during construction of RNAi vectors.**
(DOC)

Table S1 **Absolute expression data derived during qRT-PCR experiments.**
(DOC)
