# Supplementary figures and images for: RNAi Knock-Down of LHCBM1, 2 and 3 Increases Photosynthetic H2 Production Efficiency of the Green Alga Chlamydomonas reinhardtii
Source: PLoS One. 2013 Apr 16;8(4):e61375. doi: 10.1371/journal.pone.0061375 (PMC3628864; doi:10.1371/journal.pone.0061375)

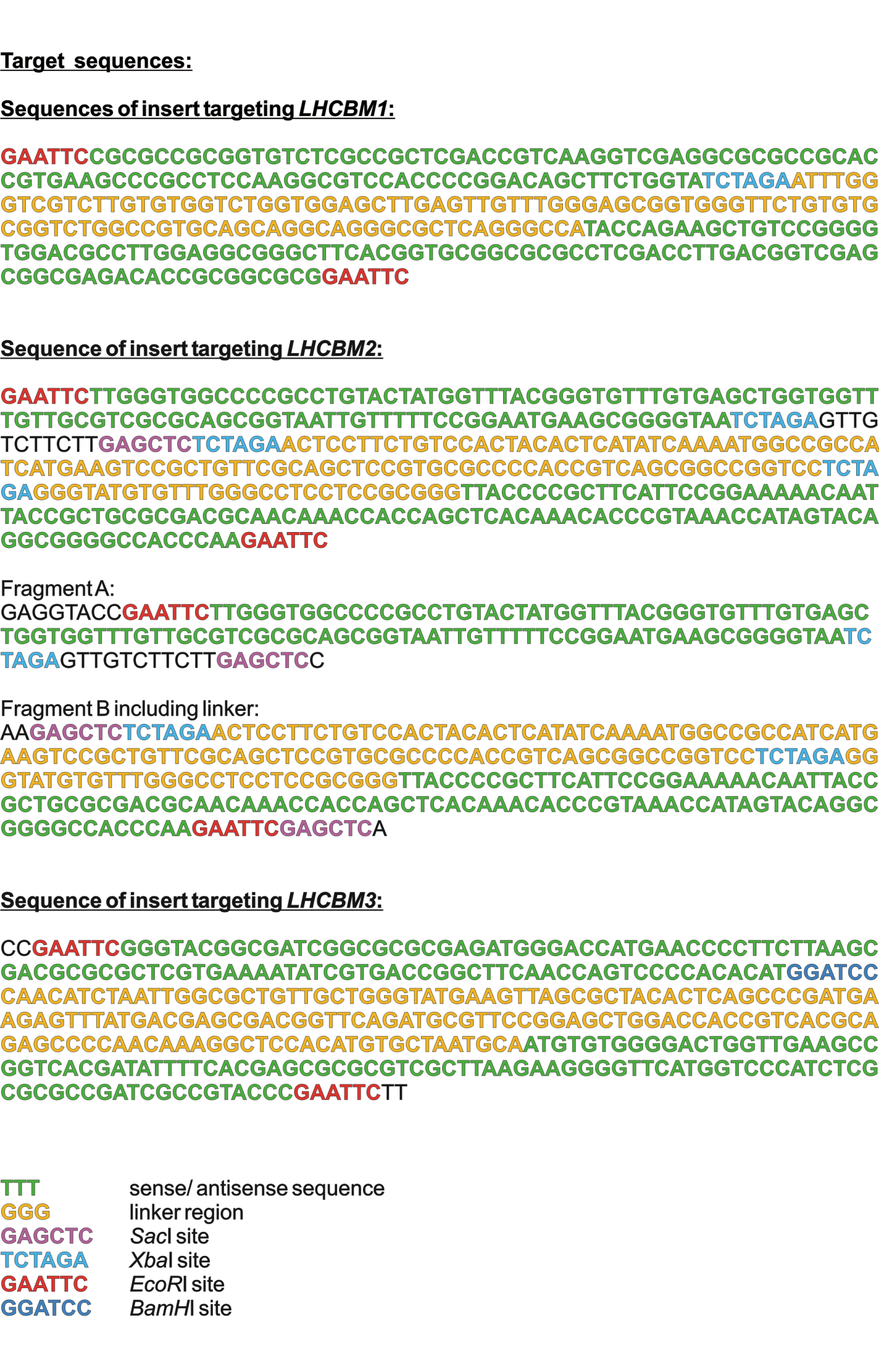

Supplement: Figure S1 — Initial RNAi oligo-nucleotides used during construction of RNAi vectors. (DOC) [file pone.0061375.s001.doc]
