# Supplementary material for: RNAi Knock-Down of LHCBM1, 2 and 3 Increases Photosynthetic H2 Production Efficiency of the Green Alga Chlamydomonas reinhardtii
Source: PLoS One. 2013 Apr 16;8(4):e61375. doi: 10.1371/journal.pone.0061375 (PMC3628864; doi:10.1371/journal.pone.0061375)
Supplement: Table S1 — Absolute expression data derived during qRT-PCR experiments. (DOC) [file pone.0061375.s002.doc]

Table S1: CT values of genes

|  | *LHCBM1* | *LHCBM2* | *LHCBM3* | *CBLP* | *18S* |
| --- | --- | --- | --- | --- | --- |
| *Stm6Glc4* | 16.757 | 19.739 | 17.883 | 18.696 | 9.88 |
|  | 16.944 | 19.933 | 17.895 | 18.91 | 9.92 |
|  | 16.935 | 19.907 | 17.948 | 18.941 | 9.923 |
| *Stm6Glc4L01* | 18.943 | 19.892 | 18.919 | 18.388 | 9.824 |
|  | 18.839 | 19.885 | 18.949 | 18.448 | 9.923 |
|  | 18.922 | 19.94 | 18.91 | 18.946 | 9.921 |
